# Modulation of brain tumor risk by genetic SNPs in PARP1gene: Hospital based case control study

**DOI:** 10.1371/journal.pone.0223882

**Published:** 2019-10-14

**Authors:** Asad ullah Khan, Ishrat Mahjabeen, Muhammad Arif Malik, Muhammad Zahid Hussain, Sarfraz Khan, Mahmood Akhtar Kayani

**Affiliations:** 1 Department of Biosciences, COMSATS University Islamabad, Islamabad, Pakistan; 2 Department of Neurosciences, Brain Surgery Hospital, Rawalpindi, Pakistan; 3 Department of Medicine, National University of Medical Sciences (NUMS), Rawalpindi, Pakistan; 4 Department of Physiotherapy, Pakistan Institute of Medical Sciences (PIMS), Islamabad, Pakistan; University of Hawaii System, UNITED STATES

## Abstract

PARP-1 gene plays an essential part in base excision repair pathway and its functional variations result in several types of cancer. In this study we have explored the effect of genetic variations in PARP-1 gene in brain tumorigenesis. This case control study comprised of 500 brain tumor cases along with 500 healthy controls. Three polymorphisms of PARP-1 gene, rs1136410 (Val762Ala), rs1805404 (Asp81Asp) and rs1805414 (Ala284Ala) were analyzed using AS-PCR method followed by DNA sequencing. Joint effect model, haplotype analysis and linkage disequilibrium of these polymorphisms was assessed using Haploview 4.2. In rs1136410 (Val762Ala) heterozygous mutant genotype (CT) was observed notably lower (OR: 0.44., 95% CI: 0.33–0.57., p<0.0001) in brain tumor patients compared to controls and ~2 fold increased frequency of homozygous mutant genotype (CC) was observed in brain tumor patients versus controls (OR: 1.51., 95%CI: 1.16–1.96, p = 0.001). In rs1805414 (Ala284Ala), frequency of heterozygous mutant genotype (CT) was observed lower (OR: 0.77., 95% CI: 0.60–0.99., p = 0.05) in patients versus controls. In rs1805404 (Asp81Asp), heterozygous mutant genotyping (CT) was observed lower in brain tumor patients compared with the healthy controls (OR: 0.63., 95% CI: 0.48–0.83., p = 0.001). However, homozygous mutant genotype (TT) was observed increased in patients compared to controls (OR: 1.41., 95% CI:1.07–1.85., p = 0.01). We assessed the fact that in combination the PARP-1 gene SNPs, rs1136410 (Val762Ala), rs1805414 (Ala284Ala) and rs1805404 (Asp81Asp) may increase the brain pathogenesis at least in Pakistani population.

## Introduction

Brain tumor refers to a collection of a new and abnormal growth of tissue presents /occurs within the bony structure called skull including brain, cranial nerves, meninges and pituitary gland etc. [1]. Brain tumors are rare but deadly since they can cause mental disability or death and are responsible for excessive mortality in children and young adults [2]. Established risk factors for brain tumors are ionizing radiation, neurofibromatosis 1, and other rare genetic syndromes [3]. Moreover, genetic susceptibility might play a pivotal role in modifying the brain tumor risk [4]. To maintain this genetic susceptibility, different DNA repair pathways perform their functions. These pathways include base excision repair pathway (BER). Any mutations in this pathway genes if left unrepaired, may lead to the process of carcinogenesis [5].

In BER pathway, Poly (ADP-ribose) polymerase 1 (PARP-1) is present on chromosome 1q41–42, comprises of 23 exons and spans 47.3 kb [6]. It codes a nuclear protein consisting both N-terminal DNA binding domain and a C-terminal catalytic domain [6]. PARP-1 gene has an important role in many cellular processes comprising DNA-damage detection and repair, cell death pathways and mitotic apparatus function [7].

Several SNPs (single nucleotide polymorphisms) have been identified in PARP-1 gene. Among these SNPs, rs1805414 (Ala284Ala) in exon 7 at position 284, lies within the PADR-1 domain. rs1805404 (Asp81Asp) in exon 2 at position 81 lies within zinc finger domain. These SNPs are associated with the risk of Alzheimer’s disease [8], glioblastoma [9], breast cancer [10] and colorectal cancer [11]. Additionally, another SNP of PARP-1 gene, rs1136410 leads to change of valine to alanine at codon 762 of catalytic domain. It reduces the activity of Poly ADP ribosylation. Up to date, several research studies have been conducted to explore the consequences of rs1136410 in several cancers such as brain, stomach, breast, colorectal, bladder and prostate cancer [12–15]. However, limited number of studies have been reported with respect to PARP-1 gene SNP analysis and brain tumors.

Present study was designed to explore the possible involvement of PARP1 gene polymorphisms in brain tumor. Additionally, the frequency of genotypes of selected PARP1 polymorphisms was also correlated with different types and grades of the brain tumor in order to further illuminate the role of these polymorphism in brain tumorigenesis.

## Materials and methods

### Study population

In present study the association of three SNPs; rs1136410 (T>C), rs1805414 (T>C) and rs1805404 (C>T), was analyzed in brain tumor patients. The study population comprised of 500 brain tumor patients along with age and sex matched 500 healthy controls collected from Nuclear Medicine Oncology & Radiotherapy Institute (NORI) (Islamabad), District Headquarter Hospital, (DHQ) (Rawalpindi), Brain Surgery Clinic (Rawalpindi), Holy Family Hospital (Rawalpindi) and Pakistan Institute of Medical Sciences (PIMS) (Islamabad). Demographic details of study cohort are given in Table 1. Inclusion criteria for control group was absence of previous cancer history, no radiation exposure. Crtieria for patient group was pathologically confirmed brain tumor by a pathologist. After obtaining consent, specifically designed questionnaire was used to collect information about demographic parameters such as smoking habits, radiation, medical and family history.

**Table 1:** Demographic characteristic of brain tumor patients and controls.

Variables	Patients (N = 500)	Controls (N = 500)	OR (95%CI)	p-value
**Age**				
Median (range)	41 (11–70)	41 (19–63)	-	-
**Gender**				
Males	319	368	-	0.05
Females	181	132		0.09
**Age**				
<41	203	191	-	0.02
≥ 41	297	309		0.01
**Smoking status**				
Smokers	191	137	1.63 (1.25–2.13)	0.0003
Non—smokers	309	363		
**Family history**				
Yes	37	6	6.57 (2.75–15.73)	< 0.0001
No	463	494		
**Ionizing radiation exposure**				
Yes	43	4	11.66 (4.15–32.76)	< 0.0001
No	457	496		
**Histological type**				
Glioma	351	-	p = 0.02	
Meningioma	149	-		
**Grading**				
Grade1	256	-	p = 0.11	
Grade2	171	-		
Grade3	67	-		
Grade4	6	-		

### Ethical approval

The study was conducted with a prior approval from the institutional ethical review board of COMSTAS University(CUI) Islamabad. Members of this committee included Dean ORIC (Office of Research Innovation and Commercialization) Prof. Dr. Raheel Qamar (convener), Prof. Dr. Mahmood A Kayani (Chairman, Deptt of Biosciences), Dr. Faheem Tahir (Deputy Director, NIH) and Dr. Tayyaba Yasmin (Associate Head of department). All samples were collected after informed consent from all participants of the study. Furthermore, the study was performed in accordance with the Declaration of Helsinki.

### SNP selections

Three functional polymorphisms of *PARP1* gene were selected using a set of web-based SNP selection tools (http://snpinfo.niehs.nih.gov/snpinfo/snpfunc.html). Following criterion was followed for selection of functional SNPs: (1) Minor allele frequency of validated SNPs > 5% in Asian population; (2) validated SNPs in important functional domain of *PARP1* gene such as Val762Ala (rs3611410, catalytic domain), Ala284Ala (rs1805414, PARD1 domain) and Asp81Asp (rs1805404, Zinc figure domain).

### DNA extraction and primer sequences for AS-PCR

Phenol Chloroform method was used for the genomic DNA isolation from white blood cells (WBCs) of blood samples of brain tumor patients and controls. Polymorphisms of PARP-1 gene were investigated by allele specific PCR (AS-PCR) using primers as given in S1 Table. PCR primers were designed by WASP (web-based allele specific primer) software http://bioinfo.biotec.or.th/WASP, by retrieving PARP1 gene sequence from ensemble with respect to polymorphism rs1136410 (T>C), rs1805414 (T>C) and rs1805404 (C>T). Two primers specified for both wild and mutant alleles were designed with a deliberate mismatch in their 2^nd^ last 3ʹ end to enhance PCR specificity. A common primer was designed upstream or downstream of the polymorphic site with no mismatch. Internal control primer of GAPDH with product length of 495 bp was used to check the reaction specificity in PCR. Primers details are given in S1 Table. All primers were checked for specific amplification using BLAST software.

### Allele specific polymerase chain reaction (AS-PCR)

An allele specific assay (AS-assay) was used for the detection of PARP-1 polymorphisms (rs1136410 (T>C), rs1805414 (T>C) and rs1805404 (C>T) in brain tumor patients and controls. Two separate PCR reactions were run in parallel, one with mutant allele primer and the other with wild type allele primers as given in S1 Table. Each PCR reaction was set out in a 10μl reaction mixture containing 1μl of genomic DNA (approximately 50ng) templates, 1μl (10mM) of each primer, 1μl nuclease free water and 4μl PCR master mix (Solis Biodyne). PCR reaction profile comprised basic denaturation step of 94°C for 5 minutes, followed by 94°C for 45 seconds, annealing temperature for 1 minute, extension at 72°C for 1 min and a final extension step of 72°C for 10 min followed by hold at 4°C.

#### Analysis of amplified products

The amplified PCR products were electrophoresed on 2% agarose gel by adding 5μl of ethidium bromide. 100bp DNA ladder (Invitrogen GeneRuler) was used for confirmation of PCR product size.

#### Sequencing

All three different patterns of alleles for wild, mutant and heterozygous genotypes were amplified in a separate reaction. Sequencing was performed by MCLab (USA). Control (normal) samples were also sequenced along with cancer cases to check the quality of sequencing.

### Statistical analysis

GraphPad prism software v 6.0. was used for the statistical investigation in the present study. Additionally, data collection of demographic parameters in the study cohort was assessed by chi-square test between the patients and controls. Hardy-Weinberg equilibrium test was performed for the actual genotypes with the expected number. Allelic frequency rate and genotyping between normal versus patients was furthered assessed by the Chi-squared tests. For calculating the odds ratios (ORs) and 95% confidence intervals (CIs), age and gender modified for the logistic regression analysis. For analyzing SNPs, three different statistical models (additive, dominant, and recessive) were performed. For SNP-SNP interactions, model of multiple logistic regression was used to explore the multiplicative interaction effect of the SNPs.

Generation of haplotypes was performed using the genotyping data. Haploview 4.2 software was used for the linkage disequilibrium (LD) and haplotype analysis using the expectation maximization (EM) algorithm.

## Results

### Genotypic frequency of selected polymorphism of PARP1 gene in study cohort

In case of rs1136410 (Val762Ala), heterozygous mutant genotype (CT) frequency was linked with 56% decrease in brain tumor risk (OR = 0.44, 95% CI: 0.33–0.57; p <0.0001). Moreover, ~2folds increase in brain tumor risk was found associated with homozygous mutant genotype (CC) (OR = 1.51, 95% CI: 1.16–1.96; p <0.002). In case of rs1805414 (Ala284Ala), heterozygous mutant genotype (CT) frequency was found associated with 23% decreased brain tumor risk (OR = 0.77, 95% CI: 0.60–0.99; p <0.05) as shown in Table 2. In rs1805404 (Asp81Asp), heterozygous mutant genotype (CT) frequency was found associated with 37% decrease in brain tumor risk (OR = 0.63, 95% CI: 0.48–0.83; p <0.001). Homozygous mutant genotype (TT) frequency was observed associated with ~1.4 folds increased risk in patients compared to controls (OR = 1.41, 95% CI: 1.07–1.85; p <0.01) as shown in Table 2.

**Table 2:** Distribution of frequency of PARP-1 SNPs in brain tumor patients and controls.

rs1136410	**Genotype / Alleles**	**Patients/Controls** **500/500** **OR; 95% CI; p-value**	**Glioma/Controls** **351/500** **OR; 95% CI; p-value**	**Meningioma/Controls** **149/500** **OR; 95% CI; p-value**
	TT	164/118 1(1)	119/118 1 (1)	45/118 1 (1)
	CT	136/229 0.44; 0.33–0.57; p < 0.0001	89/229 0.40; 0.29–0.54; p < 0.0001	47/229 0.54; 0.37–0.81; p < 0.002
	CC	200/153 1.51; 1.16–1.96; p < 0.002	143/153 1.55; 1.17–2.07; P < 0.002	57/153 1.41; 0.96–2.06; p = 0.08
	T allele frequency	464/465 1(1)	327/465 1(1)	137/465 1(1)
	C allele frequency	536/535 1.00;(0.84–1.19; p = 0.96	375/535 0.99; 0.82–1.21; p = 0.97	161/535 1.02; 0.78–1.32 p = 0.88
rs1805414	**Genotype / Alleles**	**Patients/Controls** **500/500** **OR; 95% CI; p-value**	**Glioma/Controls** **351/500** **OR; 95% CI; p-value**	**Meningioma/Controls** **149/500** **OR; 95% CI; p-value**
	TT	138/128 1(1)	105/128 1(1)	33/128 1 (1)
	CT	205/236 0.77; 0.60–0.99; p = 0.05	141/236 0.75; 0.56–0.99; P < 0.04	64/236 0.84; 0.58–1.22; p = 0.36
	CC	157/136 1.22; 0.93–1.60; p = 0.14	105/136 0.87; 0.64–1.18; P = 0.39	52/136 0.69; 0.47–1.02; P = 0.07
	T allele frequency	481/492 1(1)	351/492 1(1)	130/492 1(1)
	C allele frequency	519/508 1.04; 0.87–1.24; p = 0.62	351/508 OR:0.97; 0.79–1.17; p = 0.79	168/508 OR:1.25; 0.96–1.63; p = 0.09
rs1805404	**Genotype / Alleles**	**Patients/Controls** **500/500** **OR; 95% CI; p-value**	**Glioma/Controls** **351/500** **OR; 95% CI; p-value**	**Meningioma/Controls** **149/500** **OR; 95% CI; p-value**
	CC	208/197 1(1)	119/197 1 (1)	89/197 1 (1)
	CT	124/171 0.63; 0.48–0.83; p < 0.001	98/171 1.34; 0.99–1.80; p = 0.05	26/171 0.41; 0.25–0.64; p < 0.0001
	TT	168/132 1.41; 1.07–1.85; p < 0.01	134/132 0.58; 0.43–0.77: p < 0.0003	34/132 0.82; 0.53–1.26; p = 0.38
	C allele frequency	540/565 1(1)	336/565 1(1)	204/565 1(1)
	T allele frequency	460/435 0.90; 0.75–1.07; p = 0.26	366/435 1.41; 1.16–1.72; p < 0.0004	94/435 0.59; 0.45–0.78; p < 0.0002

Abbreviations: CI, confidence interval, OR, odds ratio, n = number, P—value.

Genotype frequency of selected polymorphisms was also calculated with different subgroups of brain cancer such as meningioma and gliomas. ~2folds increase in gliomas risk was found associated with homozygous mutant genotype (CC) (OR = 2.07, 95% CI: 1.17–2.07; p <0.002) of rs1136410 (Val762Ala). 25% decrease in gliomas risk was found linked with CT genotype of rs1805414 (OR = 0.75, 95% CI: 0.56–0.99; p <0.04) in glioma patients vs controls as shown in Table 2. In case of third selected SNP rs1805404, ~1.4folds (OR = 1.41, 95% CI: 1.16–1.72; p <0.0004) increase in glioma risk was found associated with mutant T allele in glioma vs controls (Table 2).

In case of meningioma frequency of heterozygous mutant genotype of rs1136410 was found associated with 46% decrease in meningioma (OR = 0.54, 95% CI: 0.37–0.81; p <0.002) when compared with controls. Additionally, 41% decrease in meningioma risk was also found associated with mutant T allele frequency of rs1805404 (OR = 0.59, 95% CI: 0.45–0.78; p <0.0002) in patients compared to controls as shown in Table 2.

Genotypic frequency of three selected polymorphisms of PARP1 gene was found associated with different parameters such as age, gender, smoking status, IR, types of brain tumors and grades of brain tumors by applying logistic regression model as shown in Table 3. For smoking status, only one SNP rs1136410 (OR = 2.036; 95% CI: 0.064–2.569; p <0.03) showed a positive association in brain tumor patients. Further analysis showed negative association for selected polymorphisms of PARP1 gene with other parameters such as age, gender, IR and types of brain tumors (Table 3).

**Table 3:** Association of PARP1 gene polymorphisms and different parameters of brain tumor.

SNPs vs Parameters	B	Wald	Sig.	OR	95% CI
rs1136410 vs					
Gender	0.239	1.567	0.22	0.788	0.016–3.490
Age	0.877	1.423	0.34	0.974	0.670–5.941
Smoking	**2.95**	**4.992**	**0.03**	**2.036**	**0.064–2.569**
Family History	-0.054	0.255	0.24	0.357	0.082–3.457
Ionizing radiation	0.549	1.349	0.64	0.345	1.293–4.320
Types	0.472	0.689	0.59	0.825	0.499–2.77
rs1805414 vs					
Gender	0.213	0.129	0.79	0.235	0.214–4.376
Age	0.532	0.769	0.61	1.532	0.229–8.791
Smoking	0.398	1.699	0.23	1.790	0.234–5.421
Family History	0.539	0.337	0.58	1.345	0.113–4.398
Ionizing radiation	-0.784	0.267	0.61	1.199	0.321–6.99
Types	0.229	0.189	0.22	0.645	0.129–4.339
rs1805404 vs					
Gender	0.135	0.013	0.34	0.669	0.359–2.667
Age	0.039	0.239	0.64	1.245	0.569–4.889
Smoking	1.425	0.398	0.26	0.346	0.065–6.549
Family History	1.987	1.680	0.32	1.560	0.291–4.390
Ionizing radiation	-0.391	0.005	0.92	1.491	1.233–3.290
Types	0.776	1.298	0.19	0.895	0.188–6.264

### Haplotype analysis of the *PARP1* SNPs

In the present study, haplotypes of the SNPs were constructed and analyzed for the possible association with brain tumor risk. Among these, haplotypes, CCT (OR = 0.75, 95% CI: 0.578–0.991; p <0.04) was found with significant 25% reduction in brain tumor risk (Table 4). Haplotypes were also generated and analyzed for the possible association with different subtypes of brain tumors such as glioma and meningioma. In case of gliomas total eight haplotypes were generated and among these haplotypes, CCC and TCT were observed associated with 2 folds increased risk of glioma when compared with controls, as shown in Table 4. Haplotypes CTT (OR = 0.42, 95% CI: 0.24–0.72; p <0.001) and TTT (OR = 0.54, 95% CI: 0.31–0.91; p <0.02) were found associated with 58% and 46% reduction in gliomas risk (Table 4). In case of meningioma, total eight haplotypes were generated and haplotype TTT (OR = 1.48, 95% CI: 1.04–2.09; p <0.02) was associated with 2 folds increased risk of meningioma as compared to controls, as shown in Table 4.

**Table 4:** Haplotype analysis of the PARP-1 SNPs rs1136410 (VAL762ALA), rs1805404 (Asp81Asp) and rs1805414 (Ala284Ala).

**rs1136410**	**rs1805414**	**rs1805404**	**Patients**	**Controls**	***χ***^**2**^	**OR (95% CI)**	**p-value**
C	C	C	0.172	0.158	0.649	1.10 (0.870–1.396)	0.42
C	C	T	0.107	0.137	4.133	0.75 (0.578–0.991)	0.04
C	T	C	0.130	0.148	1.313	0.86 (0.669–1.111)	0.57
C	T	T	0.131	0.122	0.376	1.08(0.834–1.414)	0.53
T	C	C	0.126	0.106	1.984	1.21 (0.925–1.603)	0.15
T	C	T	0.114	0.107	0.263	1.07 (0.814–1.423)	0.60
T	T	C	0.108	0.123	1.075	0.86 (0.657–1.138)	0.29
T	T	T	0.112	0.99	0.818	1.14(0.857–1.518)	0.36
Global result					9.300		0.23
**rs1136410**	**rs1805414**	**rs1805404**	**Glioma**	**Controls**	***χ*** ^**2**^	**OR (95% CI)**	**p-value**
C	C	C*	0.233	0.158	6.780	1.52(1.109–2.108)	0.009
C	C	T*	0.156	0.137	0.720	1.169 (0.815–1.677)	0.39
C	T	C*	0.105	0.148	3.565	0.676 (0.449–1.017)	0.05
C	T	T*	0.055	0.122	10.592	0.424 (0.249–0.721)	0.001
T	C	C*	0.137	0.106	2.156	1.336 (0.907–1.967)	0.14
T	C	T*	0.168	0.107	8.117	1.689 (1.174–2.430)	0.004
T	T	C*	0.099	0.123	1.272	0.783 (0.512–1.198)	0.25
T	T	T*	0.056	0.099	5.292	0.538 (0.315–0.919)	0.21
Global result					33.74		0.00005
**rs1136410**	**rs1805414**	**rs1805404**	**Meningioma**	**Controls**	***χ*** ^**2**^	**OR (95% CI)**	**p-value**
C	C	C*	0.124	0.158	2.715	0.752 (0.533–1.05)	0.09
C	C	T*	0.104	0.137	2.781	0.733 (0.508–1.05)	0.09
C	T	C*	0.147	0.148	0.005	0.988 (0.714–1.36)	0.94
C	T	T*	0.135	0.122	0.479	1.128 (0.802–1.58)	0.48
T	C	C*	0.098	0.106	0.208	0.914 (0.622–1.34)	0.64
T	C	T*	0.120	0.107	0.507	1.140 (0.795–1.63)	0.47
T	T	C*	0.132	0.123	0.219	1.086 (0.769–1.53)	0.64
T	T	T*	0.140	0.099	4.919	1.480 (1.045–2.09)	0.26
Global result					10.36		0.16

Abbreviations: CI, confidence interval, OR, odds ratio, n = number.

**Fig 1:**
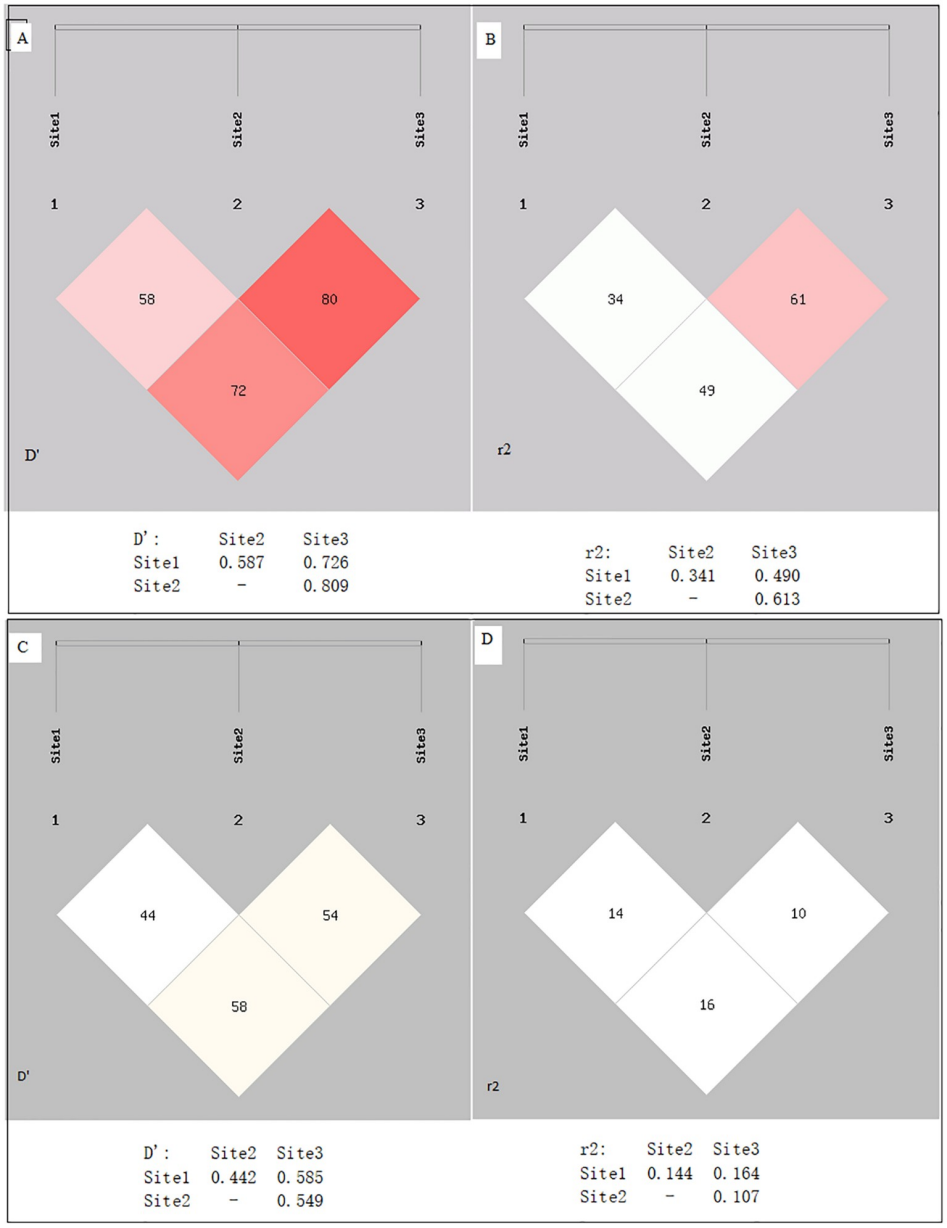
Linkage disequilibrium analysis of three selected SNPs of PARP1 gene in brain tumor patients and controls. (A) D’-value, (B) r2-value of linkage disequilibrium analysis in brain tumor patients. (C) D’-value, (D) r2-value of linkage disequilibrium analysis in controls. Site 1 for rs1136410, site 2 for rs1805414 and site 3 for rs1805404.

In addition to this, all three PARP-1 gene SNPs, rs1136410 (Val762Ala), rs1805414 (Ala284Ala) and rs1805404 (Asp81Asp) were found in strong LD in cases (Fig 1A and 1B). However, in case of controls, weak LD was observed in rs1805414 (Ala284Ala) and rs1805404 (Asp81Asp) as shown in Fig 1C and 1D.

**Fig 2:**
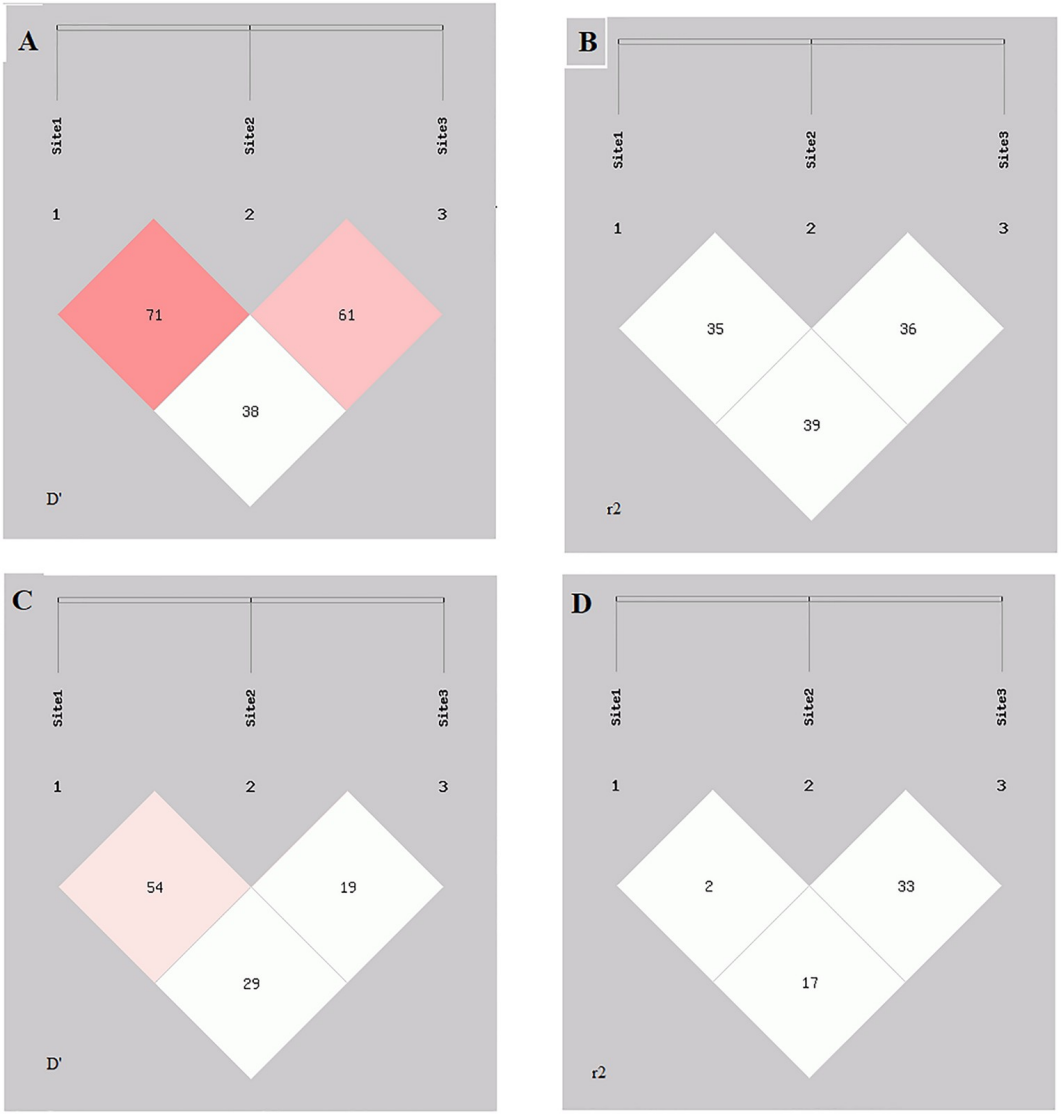
Linkage disequilibrium analysis of three selected SNPs of PARP1 gene in different subtypes of brain cancer patients. (A) D’-value, (B) r2-value of linkage disequilibrium analysis in glioma patients. (C) D’-value, (D) r2-value of linkage disequilibrium analysis in meningioma patients. Site 1 for rs1136410, site 2 for rs1805414 and site 3 for rs1805404.

Linkage disequilibrium was also calculated for selected SNPs of PARP1 gene in meningioma and glioma patients. Strong LD was found in rs1136410 (Val762Ala), rs1805414 (Ala284Ala) and rs1805404 (Asp81Asp) in glioma patients (Fig 2A and 2B). Weak LD was found in rs1136410 (Val762Ala) and rs1805414 (Ala284Ala) in meningioma patients as shown in Fig 2C and 2D.

### Combined genotype analysis of *PARP1* SNPs

Genotypic frequency of selected polymorphisms was also assessed by joint effect model. Overall, combined genotyping of three SNPs and brain tumor risk is outlined in Table 5. The *PARP1* combined genotyping SNPs findings proved statistically significant in brain tumor cases compared with controls. The patients carrying four homozygous mutant alleles showed ~3 folds increased risk of brain tumor (OR = 2.47; 95% CI: 1.16–5.22; P <0.01), ~2 folds (OR = 1.65; 95% CI = 0.98–2.76, P <0.005) higher risk in cases of patients carrying two homozygous mutant alleles and one heterozygous mutant allele and ~3 folds (OR = 2.47; 95% CI: 1.01–6.03; P <0.05) increased risk was observed in patients carrying one homozygous mutant allele (Table 5).

**Table 5:** The joint effects of SNP-SNP interactions and brain cancer risk.

rs1136410	rs1805414	rs1805404	Patients	Controls	OR (95%CI)	p–value
TT	TT	CC	16	11	1.46 (0.67–3.19)	0.33
TT	TT	CT	19	13	1.47 (0.72–3.02)	0.28
TT	TT	TT	17	07	2.47 (1.01–6.03)	0.045
TT	CT	CC	29	18	1.64 (0.90–3.00)	0.10
TT	CT	CT	19	22	0.85 (0.45–1.60)	0.63
TT	CT	TT	22	14	1.59 (0.80–3.15)	0.17
TT	CC	CC	09	15	0.59 (0.25–1.36)	0.22
TT	CC	CT	15	09	1.68 (0.73–3.89)	0.22
TT	CC	TT	18	09	2.03 (0.90–4.57)	0.08
CT	TT	CC	17	15	1.13 (0.56–2.30)	0.71
CT	TT	CT	02	19	0.10 (0.02–0.43)	0.002
CT	TT	TT	15	15	1.00 (0.48–2.06)	1.00
CT	CT	CC	21	53	0.36 (0.21–0.62)	0.0002
CT	CT	CT	11	36	0.28 (0.14–0.57)	0.0004
CT	CT	TT	15	33	0.43 (0.23–0.81)	0.00
CT	CC	CC	31	20	1.58 (0.89–2.82)	0.11
CT	CC	CT	07	25	0.26 (0.11–0.62)	0.002
CT	CC	TT	18	13	1.39 (0.67–2.88)	0.36
CC	TT	CC	21	22	0.95 (0.51–1.75)	0.87
CC	TT	CT	13	07	1.88 (0.74–4.75)	0.18
CC	TT	TT	18	18	1.00 (0.51–1.94)	1.00
CC	CT	CC	40	25	1.65 (0.98–2.76)	0.05
CC	CT	CT	27	22	1.24 (0.69–2.20)	0.46
CC	CT	TT	21	13	1.64 (0.81–3.31)	0.16
CC	CC	CC	24	18	1.35 (0.72–2.52)	0.34
CC	CC	CT	11	18	0.60 (0.28–1.28)	0.19
CC	CC	TT	24	10	2.47 (1.16–5.22)	0.01

## Discussion

PARP1 is an important BER pathway modifier in case of cellular injuries such as DNA lesion formation, strand breakage and most importantly oxidative stress [16]. Changes occur at cellular level in this gene and may trigger downstream signaling phenomena in order to facilitate DNA repair or apoptosis [6]. So far, approximately 1,066 SNPs in the PARP-1 gene have been reported among which, three SNPs; rs1136410 (Val762Ala), rs1805404 (Asp81Asp) and rs1805414 (Ala284Ala) were selected in this study for assessment of association in Pakistani population. Selection criteria for these polymorphisms was > 5% minor allele frequency in Asian population and location of selected polymorphisms in important functional domains of *PARP1* gene. To explore the association, we conducted hospital-based case control study.

For first selected polymorphism rs1136410 (Val762Ala), homozygous mutant genotype frequency was observed higher in brain tumor cases versus controls and heterozygous mutant genotype was observed lower in brain tumor cases versus controls. This is in close agreement with several other reports conducted globally, where homozygous mutant variant of PARP1 polymorphism rs1136410 was found linked with prostate cancer, thyroid carcinoma and systemic lupus erythematosus [15, 16, 17]. However, the PARP-1 (762 Ala) gene variation has also been observed playing a protective function in the initiation of a few cancers in Caucasian populations [14, 18–20]. Variant Val762Ala is located in the 6th helix of catalytic domain of the PARP-1 protein and replacing valine with alanine causes a decrease in PARP-1 enzyme activity [21] and its interaction with other scaffold proteins of BER pathway gene, such as XRCC1 [22], ultimately results in increased frequency of DNA damage and oxidative load in brain cells.

Heterozygous mutant genotype of second selected SNP of PARP1 gene rs1805414, showed protective effect against the brain tumor risk in Pakistani population. Besides that, rs1805414 is located in exon 7 at position 284, lies within the PADR1 domain (Clade 1 PARPs) but its function is still unknown. Its association has been established with an increased risk of alzheimer’s disease [8], glioblastoma [23], breast cancer [10] and decreased risk with colorectal cancer [11, 24]. Milani et al., (2007) has reported that rs1805414 is located in promoter region of PARP1 gene and the expression level of this gene is altered by allelic imbalance in cancerous cells [25].

In SNP rs1805404 (Asp81Asp), heterozygous mutant genotype showed the protective association against brain carcinogenesis and homozygous mutant genotype was found higher in brain tumor cases compared with controls. Limited number of studies has been reported for the said SNP with contradictory trend where mutant allele of Asp81Asp has shown protector effect against cancer [26]. rs1805404 is found located in zinc finger domain of PARP1 gene, important for recognition of DNA damage [27] and this variant may result in accumulation of un-repaired damage and increased mutational load in brain tissue.

These SNPs were correlated with different parameters such as type and grades of brain tumor and increased frequency of mutant genotypes were observed in advanced grades of brain tumors. Thus, on the basis of these observations we hypothesize that selected SNPs are strongly involved in biology and oncogenesis of brain tumorigenesis. Several earlier studies have reported increased risk of genetic variations of BER pathway genes in advanced grade brain tumor [28–30]. Furthermore, similar trends have also been observed in the frequency of gene variations in gliomas and grades of brain tumors [31–33].

Haplotypes were generated of the three SNPs for the PARP1 gene and haplotype CCT was observed more in controls versus brain tumor cases. Other generated haplotypes were not found associated with brain tumorigenesis. One of the possible reasons of this weak association may be the linkage disequilibrium with other functional variants in PARP1 gene. To our knowledge, no prior findings have observed the cumulative effect of Val762Ala (rs1136410), Ala284Ala (rs1805414) and Asp81Asp (rs1805404) SNPs of PARP1 gene in brain tumor. *PARP1* gene is polymorphic in nature and in present study we selected only three SNPs. Further studies on other functional *PARP1* SNPs are needed to define the role of the *PARP1* gene polymorphisms in brain tumor.

Haplotypes were also generated for subtypes of different brain tumors such as glioma and meningioma. 2-fold increased risk of developing gliomas was observed by combining three putative risk genotypes. Present study also showed that selected SNPs has significant increased involvement in glioma compared to meningioma pathogenesis. Collectively, it is suggested that a combined interaction among the susceptibility genotypes is in line with the poly-allelic model, in which many alleles confer susceptibility in the population. Low penetrating variations (like SNPs) usually alter the cancer susceptibility, but the potential of these variations often lie within their synergistic domain which is much effective [34].

Selected SNPs of PARP1 gene were linked with the overall risk of brain tumor mutant allele of rs1136410 and rs1805404 SNPs were found involved in increased risk of brain tumor. This increased risk of brain tumor was more pronounced in combined genotype effect. We assessed the fact that these SNPs might increase the brain pathogenesis in combination, which is in accordance with mutagenic nature of carcinogenesis. This shows that alterations in multiple steps of BER pathway will more affect the brain tumor risk than alterations in a single step. Moreover, the heterozygotes of each selected polymorphism has a lower risk of brain tumor. Cells with heterozygous PARP-1 may have a wider range of molecular specificity for base excision repair, therefore preventing the tumorigenesis of brain tumors more effectively.

There are many potential limitations in the present study which need to be considered. Firstly, a large sample size in the future studies (with various ethnic backgrounds) may be used to further confirm the association between SNPs of PARP-1 gene and brain tumor susceptibility. Secondly, subjects under investigation in this case-control association study came from two hospitals and it may lead towards the selection bias that may have profound effect on the present research findings. Consequently, studies involving larger set of data are recommended to validate these findings.

## Supporting information

S1 Table Primers designed for PARP-1 polymorphisms rs1136410 (T>C), rs1805414 (T>C) and rs1805404 (C>T) with their product lengths and optimizing temperatures. (DOCX)

## References

[1] Hill JR, Kuriyama N, Kuriyama H, Israel MA. Molecular genetics of brain tumors. Archives of neurology. 1999 4 1;56(4):439–41. 10.1001/archneur.56.4.439 10199332

[2] KK K, Rajan MS, Hegde K, Koshy S, Shenoy A. A comprehensive review in brain tumor. International Journal of Pharmaceutical, Chemical and Biological Science. 2013 2 3; 3 (4):1165–1171.

[3] Crump C, Sundquist J, Sieh W, Winkleby MA, Sundquist K. Perinatal and familial risk factors for brain tumors in childhood through young adulthood. Cancer research. 2015 1;75(3):576–83. 10.1158/0008-5472.CAN-14-2285 25511376 PMC4315700

[4] Bohr VA, Ottersen OP, Tønjum T. Genome instability and DNA repair in brain, ageing and neurological disease. Neuroscience. 2007; 145 (4):1183–6. 10.1016/j.neuroscience.2007.03.015 . 17400394

[5] Bethke L, Webb E, Murray A, Schoemaker M, Johansen C, Christensen HC, et al Comprehensive analysis of the role of DNA repair gene polymorphisms on risk of glioma. Human molecular genetics. 2007 11 29;17(6):800–5. 10.1093/hmg/ddm351 . 18048407

[6] Schreiber V, Dantzer F, Ame JC, De Murcia G. Poly (ADP-ribose): novel functions for an old molecule. Nature reviews Molecular cell biology. 2006 7;7(7):517 10.1038/nrm1963 16829982

[7] Kim MY, Zhang T, Kraus WL. Poly (ADP-ribosyl) ation by PARP-1:PAR-laying’NAD+ into a nuclear signal. Genes & development. 2005 9 1;19(17):1951–67. 10.1101/gad.1331805 . 16140981

[8] Liu HP, Lin WY, Wu BT, Liu SH, Wang WF, Tsai CH, et al Evaluation of the poly (ADP‐ribose) polymerase‐1 gene variants in Alzheimer’s disease. Journal of clinical laboratory analysis. 2010 1 1;24(3):182–6. 10.1002/jcla.20379 . 20486200 PMC6647671

[9] Yosunkaya E, Kucukyuruk B, Onaran I, Gurel CB, Uzan M, Kanigur-Sultuybek G. Glioma risk associates with polymorphisms of DNA repair genes, XRCC1 and PARP1. British journal of neurosurgery. 2010 10 1;24(5):561–5. 10.3109/02688697.2010.489655 . 20868244

[10] Alanazi M, Pathan AA, Shaik JP, Al Amri A, Parine NR. The C Allele of a synonymous SNP (rs1805414, Ala284Ala) in PARP1 is a risk factor for susceptibility to breast cancer in Saudi patients. Asian Pacific Journal of Cancer Prevention. 2013;14(5):3051–6. 10.7314/apjcp.2013.14.5.3051 . 23803078

[11] Berndt SI, Huang WY, Fallin MD, Helzlsouer KJ, Platz EA, Weissfeld JL, et al Genetic variation in base excision repair genes and the prevalence of advanced colorectal adenoma. Cancer research. 2007 2 1;67(3):1395–404. 10.1158/0008-5472.CAN-06-1390 . 17283177

[12] Yosunkaya E, Kucukyuruk B, Onaran I, Gurel CB, Uzan M, Kanigur-Sultuybek G. Glioma risk associates with polymorphisms of DNA repair genes, XRCC1 and PARP1. British journal of neurosurgery. 2010 10 1;24(5):561–5. 10.3109/02688697.2010.489655 . 20868244

[13] Li C, Hu Z, Lu J, Liu Z, Wang LE, El‐Naggar AK, et al Genetic polymorphisms in DNA base‐excision repair genes ADPRT, XRCC1, and APE1 and the risk of squamous cell carcinoma of the head and neck. Cancer. 2007 8 15;110(4):867–75. 10.1002/cncr.22861 . 17614107

[14] Qin Q, Lu J, Zhu H, Xu L, Cheng H, Zhan L, et al PARP-1 Val762Ala polymorphism and risk of cancer: a meta-analysis based on 39 case-control studies. PloS one. 2014 5 22;9(5):e98022 10.1371/journal.pone.0098022 24853559 PMC4031170

[15] Lockett KL, Hall MC, Xu J, Zheng SL, Berwick M, Chuang SC, et al The ADPRT V762A genetic variant contributes to prostate cancer susceptibility and deficient enzyme function. Cancer research. 2004 9 1;64(17):6344–8. 10.1158/0008-5472.CAN-04-0338 . 15342424

[16] Chiang FY, Wu CW, Hsiao PJ, Kuo WR, Lee KW, Lin JC, et al Association between polymorphisms in DNA base excision repair genes XRCC1, APE1, and ADPRT and differentiated thyroid carcinoma. Clinical Cancer Research. 2008 9 15;14(18):5919–24. 10.1158/1078-0432.CCR-08-0906 18779313

[17] Hur JW, Sung YK, Shin HD, Park BL, Cheong HS, Bae SC. Poly (ADP-ribose) polymerase (PARP) polymorphisms associated with nephritis and arthritis in systemic lupus erythematosus. Rheumatology. 2006 2 3;45(6):711–7. 10.1093/rheumatology/kei262 . 16461442

[18] Cottet F, Blanché H, Verasdonck P, Le Gall I, Schächter F, Bürkle A, et al New polymorphisms in the human poly (ADP-ribose) polymerase-1 coding sequence: lack of association with longevity or with increased cellular poly (ADP-ribosyl) ation capacity. Journal of molecular medicine. 2000 10 1;78(8):431–40. . 11097112 10.1007/s001090000132

[19] Berndt SI, Huang WY, Fallin MD, Helzlsouer KJ, Platz EA, Weissfeld JL et al Genetic variation in base excision repair genes and the prevalence of advanced colorectal adenoma. Cancer research. 2007; 67(3):1395–404. 10.1158/0008-5472.CAN-06-1390 17283177

[20] Li C, Liu Z, Wang LE, Strom SS, Lee JE, Gershenwald JE, et al Genetic variants of the ADPRT, XRCC1 and APE1 genes and risk of cutaneous melanoma. Carcinogenesis. 2006 4 18;27(9):1894–901. 10.1093/carcin/bgl042 . 16621887

[21] Higgins JP, Thompson SG, Deeks JJ, Altman DG. Measuring inconsistency in meta-analyses. BMJ: British Medical Journal. 2003 9 6;327(7414):557 10.1136/bmj.327.7414.557 12958120 PMC192859

[22] Keller A, Harz C, Matzas M, Meder B, Katus HA, Ludwig N, et al Identification of novel SNPs in glioblastoma using targeted resequencing. PloS one. 2011 6 10;6(6):e18158 10.1371/journal.pone.0018158 21695249 PMC3112142

[23] Ogino S, Stampfer M. Lifestyle factors and microsatellite instability in colorectal cancer: the evolving field of molecular pathological epidemiology. J Natl Cancer Inst. 2010; 102(6):365–7. 10.1093/jnci/djq031 20208016 PMC2841039

[24] Milani L, Gupta M, Andersen M, Dhar S, Fryknäs M, Isaksson A, et al Allelic imbalance in gene expression as a guide to cis-acting regulatory single nucleotide polymorphisms in cancer cells. Nucleic acids research. 2007 1 31;35(5):e34 10.1093/nar/gkl1152 17267408 PMC1865061

[25] Shiokawa M, Masutani M, Fujihara H, Ueki K, Nishikawa R, Sugimura T, et al Genetic alteration of poly (ADP-ribose) polymerase-1 in human germ cell tumors. Japanese journal of clinical oncology. 2005 2 1;35(2):97–102. 10.1093/jjco/hyi028 15709096

[26] Ali AA, Timinszky G, Arribas-Bosacoma R, Kozlowski M, Hassa PO, Hassler M, et al The zinc-finger domains of PARP1 cooperate to recognize DNA strand breaks. Nature Structural and Molecular Biology. 2012 7;19(7):685 10.1038/nsmb.2335 22683995 PMC4826610

[27] Kiuru A, Lindholm C, Heinävaara S, Ilus T, Jokinen P, Haapasalo H, et al XRCC1 and XRCC3 variants and risk of glioma and meningioma. Journal of neuro-oncology. 2008 6 1;88(2):13542 10.1007/s11060-008-9556-y . 18330515

[28] Wang LE, Bondy ML, Shen H, El-Zein R, Aldape K, Cao Y, et al Polymorphisms of DNA repair genes and risk of glioma. Cancer research. 2004 8 15;64(16):5560–3. 10.1158/0008-5472.CAN-03-2181 . 15313891

[29] Malmer BS. Feychting M, Lönn S, Lindström S, Grönberg H, Ahlbom A, et al Genetic variation in p53 and ATM haplotypes and risk of glioma and meningioma. J Neurooncol. 2007;82(3):229–37. 10.1007/s11060-006-9275-1 17151932

[30] Malmer BS, Feychting M, Lönn S, Lindström S, Grönberg H, Ahlbom A, et al Genetic variation in p53 and ATM haplotypes and risk of glioma and meningioma. Journal of neuro-oncology. 2007 5 1;82(3): 229–37. 10.1007/s11060-006-9275-1 . 17151932

[31] Juratli TA, Kirsch M, Robel K, Soucek S, Geiger K, von Kummeret al. IDH mutations as an early and consistent marker in low-grade astrocytomas WHO grade II and their consecutive secondary high-grade gliomas. Journal of neuro-oncology. 2012 7 1;108(3):403–10. 10.1007/s11060-012-0844-1 22410704

[32] Thota B, Shukla SK, Srividya MR, Shwetha SD, Arivazhagan A, Thennarasu K, et al IDH1 mutations in diffusely infiltrating astrocytomas: grade specificity, association with protein expression, and clinical relevance. American journal of clinical pathology. 2012 8 1;138(2):177–84. 10.1309/AJCPZOIY3WY4KIKE 22904127

[33] Lewandowska MA, Furtak J, Szylberg T, Roszkowski K, Windorbska W, Rytlewska J, et al An analysis of the prognostic value of IDH1 (isocitrate dehydrogenase 1) mutation in Polish glioma patients. Molecular diagnosis & therapy. 2014 2 1;18(1):45–53. 10.1007/s40291-013-0050-7 23934769 PMC3899509

[34] Chiang FY, Wu CW, Hsiao PJ, Kuo WR, Lee KW, Lin JC, et al Association between polymorphisms in DNA base excision repair genes XRCC1, APE1, and ADPRT and differentiated thyroid carcinoma. Clinical Cancer Research. 2008 9 15;14(18):5919–24. 10.1158/1078-0432.CCR-08-0906 18779313

