# Supplementary material for: Modulation of brain tumor risk by genetic SNPs in PARP1gene: Hospital based case control study
Source: PLoS One. 2019 Oct 14;14(10):e0223882. doi: 10.1371/journal.pone.0223882 (PMC6791555; doi:10.1371/journal.pone.0223882)
Supplement: S1 Table — (DOCX) [file pone.0223882.s001.docx]

**S1 Table. Primers designed for PARP-1 polymorphisms rs1136410 (T>C), rs1805414 (T>C) and rs1805404 (C>T) with their product lengths and optimizing temperatures**

| **Gene (PARP-1)** | **Primer sequence** | **Product length(bp)** | **Optimizing temp ^o^C** |
| --- | --- | --- | --- |
| rs1136410 (T>C) | Reverse primer (wild) TTGCTCCTCCAGGCCAAGTC | 236bp | 60^o^C |
|  | Reverse primer (recessive) TTGCTCCTCCAGGCCAAGTT |  |  |
|  | Common forward primer CAGCTTTCCAGGAGATCCTA |  |  |
| rs1805414 (T>C) | Forward primer (wild) GCAGATCTTGGACCGAGTAGAC | 253 bp | 55^o^C |
|  | Forward primer (recessive) GCAGATCTTGGACCGAGTAGAT |  |  |
|  | Common reverse primer GGTGTCTGTGTCTTGACCAT |  |  |
| rs 1805404 (C>T) | Forward primer (wild) TGAGCTTCGGTGGGATGTC | 213bp | 56^o^C |
|  | Forward primer (recessive) TGAGCTTCGGTGGGATGTT |  |  |
|  | Common reverse primer GTGTTGCTGAAATAACATGG |  |  |
| GAPDH  (Internal Control) | Forward Primer CAAGGTCATCCATGACAACTTTG | 495 bp | 58 ^o^C |
|  | Reverse Primer GTCCACCACCCTGTTGCTGTAG |  |  |

Underlined base is deliberate mismatch inserted in primer sequence.
